# Cognitive decline and risk of all-cause mortality in older women: a cohort study

**DOI:** 10.1186/s12877-026-07641-1

**Published:** 2026-05-13

**Authors:** Ricarda S. Schulz, Toivo Glatz, Julie E. Buring, Jae H. Kang, Tobias Kurth, Pamela M. Rist

**Affiliations:** 1 Institute of Public Health, Charité – Universitätsmedizin Berlin, Freie Universität Berlin and Humboldt-Universität zu Berlin, Charitéplatz 1, Berlin, 10117 Germany; 2 Division of Preventive Medicine, Department of Medicine, Brigham and Women’s Hospital, Harvard Medical School, Boston, MA USA; 3 Channing Division of Network Medicine, Department of Medicine, Brigham and Women’s Hospital, Harvard Medical School, Boston, MA USA

**Keywords:** Cognition, Cognitive decline, Mortality, Women, Women’s Health, Cohort Study, Epidemiology

## Abstract

**Background:**

With global population aging and an increasing prevalence of cognitive decline, the association between cognitive change as a dynamic process and all-cause mortality remains insufficiently investigated. We therefore aimed to assess the association of cognitive decline on all-cause mortality in older women.

**Methods:**

We analyzed data originating from the cognitive cohort of the Women’s Health Study established in 1998. 6,377 US-based women aged ≥ 65 years and currently or formerly employed in health-related professions were enrolled at baseline. Women with complete information on 4-year global cognitive performance change were eligible for our analysis and assigned to quintiles according to change in global cognitive performance. Follow-up for all-cause mortality was administered until December 31, 2022. Multivariable adjusted Cox proportional hazards regression models were used to estimate hazard ratios (HRs) and 95% CIs of the effect of cognitive change on the risk of all-cause mortality. Secondary analyses evaluated the risk in the lowest 20% and 10% of the cognitive change distribution, and according to verbal memory changes.

**Results:**

5,214 women with a mean age of 66.1 years (SD 4.0) were included in the analyses. Women were followed for an average of 13.4 person years and 3,333 deaths were observed. When compared to the 5th quintile with the greatest improvement, the adjusted HR for all-cause mortality was 0.96 (95%CI [0.86–1.08]) for the 4th, 1.04 (95%CI [0.93–1.16]) for the 3rd, 1.15 (95%CI [1.04–1.29]) for the 2nd, and 1.37 (95%CI [1.23–1.52]) for the 1st quintile characterized by the greatest decline. Similar patterns were observed for verbal memory change. Risk was further elevated when comparing the worst 20% (HR: 1.32 (95%CI [1.21–1.43])) and 10% (HR: 1.44 (95%CI [1.29–1.60])) to all other participants.

**Conclusion:**

Cognitive decline over four years was associated with an increased risk of mortality among older women. Further studies should explore whether declines earlier in life or among men are also associated with an increased risk of mortality.

**Supplementary Information:**

The online version contains supplementary material available at 10.1186/s12877-026-07641-1.

## Introduction

Cognitive decline is a major determinant of loss of independence, increased care needs, and diminished quality of life in older adults, and contributes substantially to the burden on health systems and society [1–3]. As populations age globally, understanding the downstream consequences of cognitive decline, including its potential association with mortality, has become increasingly relevant and preserving cognitive function is widely regarded as a cornerstone of healthy aging [4, 5].

Several population-based studies have investigated the association between cognitive impairment, or less frequently, cognitive decline and mortality risk, suggesting that poorer cognitive function might elevate the risk for all-cause mortality [6–9].

Cognitive decline and mortality may share common pathophysiological processes, with poor vascular health contributing to both brain pathology and cerebrovascular events [10–12].

More recently there is growing interest in potential sex-differences in these associations. Population-based studies from Germany, Denmark, and Brazil found that cognitive impairment was more strongly associated with mortality in men than in women, even at similar levels of impairment and after adjusting for factors such as education, demographics, and other health and lifestyle factors [13–15]. In contrast, a recent meta-analysis on the sex-specific impact of cognitive impairment on all-cause mortality in elderly people reported an elevated risk for all-cause mortality, with women being at higher risk compared to men [6].

Despite previous research being conducted on the effect of cognitive impairment on all-cause mortality, fewer studies have examined the association between patterns of cognitive change over time and mortality risk. Examining cognitive change may allow for a more nuanced understanding of the association between cognitive function and mortality. For example, individuals who demonstrate high cognitive performance and continue on a relatively high-functioning trajectory, despite showing a measurable decline in cognitive abilities, would not be considered to be cognitive impaired in analyses which only focus on cross-sectional measures of cognitive function. However, this process of decline may reflect underlying pathology that may increase the risk of mortality. While some studies have observed associations between cognitive decline and mortality, few studies have specifically examined this association among women, who may have faster rates of cognitive decline than men [8, 16].

To further add to the literature on cognitive decline and mortality risk, particularly among women, we aimed to analyze the effect of cognitive decline over a about four-year period on all-cause mortality within a large prospective cohort of US-based women 65 years and older at baseline.

## Methods

### Study population

The population under study was composed of women participating in the cognitive cohort of the Women’s Health Study (WHS). The WHS (ClinicalTrials.gov Identifier: NCT00000479; study registration date: first posted (estimated) October 28th, 1999) was designed as a randomized, placebo-controlled trial to assess the effects of low-dose aspirin and vitamin E in the primary prevention of cardiovascular disease (CVD) and cancer in women. More nuanced information on the study’s design, methods, and results have previously been reported [17, 18].

In summary, 39,876 women living in the United States, employed in health professions, at least 45 years of age, and free of a history of CVD, cancer, and other major diseases were randomized into the study (1992–1995) [19]. The main trial ended on March 31st, 2004 and observational follow-up is ongoing.

Written informed consent was provided by all women participating in the WHS. The WHS received approval from the Institutional Review Board of the Brigham and Women’s Hospital, Boston, MA (IRB number observational follow-up: 2004P001661).

### Population for analysis and follow-up

In 1998, a cognitive cohort was established into the WHS (IRB number cognitive cohort: 1999P002903). Women who were at least 65 years of age and actively participating in the main WHS trial were eligible for inclusion into the cohort (*N* = 7,175). Of those eligible, 6,377 (88.9%) women completed an initial cognitive assessment by telephone [20]. In this study, we focused on changes from the baseline assessment to the last cognitive assessment performed approximately four years later and excluded women who did not participate in the last cognitive assessment. Women with incomplete data for any cognitive test at either the first or last cognitive assessment required for the computation of the global cognitive performance change were also excluded (eFigure1).

### Assessment of cognitive performance

Cognitive function was assessed via a test battery composing five separate cognitive tests which were administered by trained nurses [21]. The test battery included: (1) The Telephone Interview of Cognitive Status (TICS), which is a telephone adaption of the Mini Mental State Examination [22] (MMSE) measuring general cognition and reported to be valid and reliable by previous research [23]. The score range for this test ranges from 0 to 41 points. (2) Immediate and (3) delayed recalls of the East Boston Memory Test which measure verbal memory. The score range for these tests are 0 to 12 [20, 21]. (4) The TICS 10-word list was further used to measure delayed verbal memory with a score range from 0 to 10. (5) Lastly, women were asked to name as many animals as they could within a minute to assess category fluency, including language and executive functioning [20, 24].

We averaged the *z*-score from each test at each time point separately based on the distribution of scores at the first assessment to define global cognitive performance change. This approach has been previously applied for the analysis of WHS data [21]. We then classified women by assigning them into quintiles based on the distribution of the change in *z*-score between the first and last cognitive assessment four years later.

### Assessment of all-cause mortality

The outcome of interest was all-cause mortality. Deaths were identified by reports from family members, next of kin, postal authorities, or the National Death Index searches. Information on the date of death was ascertained from medical records, death certificates or the National Death Index (NDI). The NDI is a centralized database maintained by the US National Center for Health Statistics providing information on deaths including mortality status and cause of death within the US.

### Statistical analysis

#### Descriptive analysis of the study cohort

We report mean values with standard deviations (SDs) for continuous data and frequency proportions for categorical variables for demographic characteristics at baseline by quintile of global cognitive performance change. Additionally, we report the mean test performance for each test administered at each timepoint by quintile of global cognitive change.

To assess potential bias due to differential loss to follow-up, we further compared baseline characteristics and cognitive test performance of women who participated in the first but not in the last cognitive assessment to those who participated in both assessments.

#### Primary analysis

Person-time was calculated from the last cognitive assessment until death, loss to follow-up, or the present study’s end date (administrative censoring on December 31, 2022), whichever was encountered first.

We visually assessed Kaplan-Meier curves to detect violations of the proportional hazards assumption and conducted log-rank tests to evaluate overall differences between survival curves.

To estimate unadjusted and adjusted hazard ratios (HRs) with corresponding 95% CIs for the association between cognitive change and the risk of all-cause mortality, we applied Cox proportional hazards regression models [25] using the 5th quintile, i.e., the quintile with the greatest improvement, as the reference.

We adjusted the statistical models for potential confounders of the effect of cognitive change on the risk of all-cause mortality. The variable selection into the models’ adjustment set was informed by expert knowledge and a review of relevant literature [26, 27]. To illustrate the adjustment strategy, we created a Directed Acyclic Graph (DAG) utilizing the DAGitty [28] software (eFigure 2).

We adjusted our models for the subsequent information reported at study entry: age (in years), highest attained education (Licensed Practical Nurse/Licensed Vocational Nurse, 2-yr Assoc./Registered Nurse, 3-yr Assoc./Registered Nurse, Bachelor, Master, Doctorate/MD), Body Mass Index (BMI) (in kg/m²), strenuous physical activity (rarely/never, < 1 time/week, 1 time/week, 2–3 timer/week, 4–6 times/week, 7 + times/week), smoking status (never, current, past), alcohol use (rarely/never, 1–3 drinks/month, 1–6 drinks/week, 1 + drinks/day), and prior history (yes/no) of each of the following: diabetes, hypertension, treatment for high blood pressure, high cholesterol, and treatment for high cholesterol.

We assessed potential multicollinearity among adjustment variables using generalized variance inflation factors (GVIF). All GVIF^(1/(2*Df)) values were close to 1, indicating no evidence of problematic multicollinearity in our Cox proportional hazards regression model.

Additionally, we estimated the restricted mean survival time (RMST) comparing women in the bottom 20% of the cognitive decline distribution against the rest of the population. The RMST represents the average survival time from baseline up until a pre-defined truncation time τ which was set to 19.9 years (maximum follow up for the two groups compared) and does not rely on the proportional hazards assumption.

#### Secondary analyses

We further aimed to estimate the effect of cognitive decline on all-cause mortality for women in the bottom 20% and 10% of the cognitive change distribution compared to the rest of the sample. We applied the same statistical modeling procedures, including the adjustment set, as specified for the primary analysis.

Additionally, we re-categorized the women into verbal memory change quintiles by computing average differences in the *z*-scores between the first and last cognitive interview exclusively considering verbal memory tests scores (immediate and delayed recalls of the East Boston Memory Test and the delayed recall of TICS 10 word-list). This analysis was performed as verbal memory is a strong predictor of Alzheimer’ Disease (AD), the leading cause of dementia [29], and AD is put forward as a risk factor for mortality [30, 31]. This procedure has been applied in the context of the cognitive cohort of the WHS previously [21].

#### Handling of missing data

Across all variables included in the models, the proportion of missing values did not exceed 1.6% for each variable. For missing BMI values, the mean was imputed. Categorical variables were imputed by assigning the most common response for each: ‘*rarely/never*’ for both alcohol consumption and physical activity; ‘*3-year Associate/RN*’ for educational attainment; and ‘*no*’ for baseline history of diabetes, hypertension, treatment for high blood pressure, history of high cholesterol, and treatment for high cholesterol. Those missing information on smoking status were assigned to ‘*past user*’.

Additionally, we conducted a sensitivity analysis using multiple imputation by chained equations repeating our primary analysis. Five imputed datasets were generated and Cox proportional hazards regression models were fitted within each imputed dataset and estimates were pooled.

A type I error of 5% was defined a priori and we considered *p*-values ≤ 0.05 as statistically significant in a two-tailed test. We performed our statistical analysis using the statistical software R [32] (version 4.5.2) and RStudio (version 2024.09.1).

## Results

Following the exclusion of those women who did not take part in both (first and last) cognitive assessment (*n* = 1,151) and those with missing data in at least one cognitive test required to calculate global cognitive change between the first and last assessment (*n* = 12), a total of 5,214 women were eligible for our analyses.

We did not observe major differences between demographic characteristics by global cognitive change quintiles (see Table 1). The mean overall age at baseline was 66.1 (SD 4.0) years with 33.0% of the cohort reporting a bachelor’s degree or higher. 46.5% of the women indicated that they rarely or never consume alcohol, and 52.9% report that they have never smoked. With respect to physical activity, 42.4% of the women reported that they seldom or never engage in strenuous exercises. In terms of medical history, 39.2% of the women reported a history of hypertension, and 43.4% reported a history of high cholesterol.

**Table 1:** Baseline characteristics of eligible women in the WHS cognitive cohort by global cognitive performance quintiles

	1st quintile (*n* = 1,043)	2nd quintile (*n* = 1,043)	3rd quintile (*n* = 1,042)	4th quintile (*n* = 1,043)	5th quintile (*n* = 1,043)	Overall (*N* = 5,214)
Age						
Mean (SD)	67.0 (4.32)	66.1 (4.00)	65.8 (3.89)	65.8 (3.74)	65.6 (3.57)	66.1 (3.95)
Median [Min, Max]	66.5 [60.5, 83.1]	65.3 [60.5, 87.1]	65.0 [60.4, 82.8]	65.1 [60.5, 82.3]	64.8 [60.4, 80.2]	65.4 [60.4, 87.1]
BMI						
Mean (SD)	25.7 (4.40)	25.9 (4.59)	25.8 (4.51)	25.9 (4.29)	25.7 (4.42)	25.8 (4.44)
Median [Min, Max]	24.9 [17.0, 47.5]	25.2 [15.7, 48.0]	25.0 [16.0, 45.3]	25.1 [17.7, 51.5]	25.0 [16.1, 48.4]	25.1 [15.7, 51.5]
Missing	1 (0.1%)	0 (0%)	0 (0%)	0 (0%)	0 (0%)	1 (0.0%)
Highest attained education						
LPN/LVN, associate’s degree, registered nurse	678 (65.0%)	662 (63.5%)	693 (66.5%)	679 (65.1%)	695 (66.6%)	3,407 (65.3%)
Bachelor’s degree or higher education	352 (33.7%)	365 (35.0%)	336 (32.2%)	340 (32.6%)	330 (31.6%)	1,723 (33.0%)
Missing	13 (1.2%)	16 (1.5%)	13 (1.2%)	24 (2.3%)	18 (1.7%)	84 (1.6%)
Smoking status						
Never	546 (52.3%)	550 (52.7%)	549 (52.7%)	557 (53.4%)	555 (53.2%)	2,757 (52.9%)
Past	398 (38.2%)	396 (38.0%)	394 (37.8%)	401 (38.4%)	387 (37.1%)	1,976 (37.9%)
Current	96 (9.2%)	97 (9.3%)	98 (9.4%)	84 (8.1%)	100 (9.6%)	475 (9.1%)
Missing	3 (0.3%)	0 (0%)	1 (0.1%)	1 (0.1%)	1 (0.1%)	6 (0.1%)
Strenuous physical activity						
Rarely/never	443 (42.5%)	458 (43.9%)	413 (39.6%)	451 (43.2%)	448 (43.0%)	2,213 (42.4%)
<1 time/week	159 (15.2%)	193 (18.5%)	179 (17.2%)	171 (16.4%)	157 (15.1%)	859 (16.5%)
1 time per week	95 (9.1%)	74 (7.1%)	87 (8.3%)	75 (7.2%)	100 (9.6%)	431 (8.3%)
2–3 times/week	206 (19.8%)	207 (19.8%)	246 (23.6%)	219 (21.0%)	215 (20.6%)	1,093 (21.0%)
≥ 4 times/week	138 (13.2%)	110 (10.5%)	115 (11.0%)	127 (12.2%)	123 (11.8%)	613 (11.8%)
Missing	2 (0.2%)	1 (0.1%)	2 (0.2%)	0 (0%)	0 (0%)	5 (0.1%)
Alcohol use						
Rarely/never	494 (47.4%)	489 (46.9%)	475 (45.6%)	486 (46.6%)	479 (45.9%)	2,423 (46.5%)
1–3 drinks/month	124 (11.9%)	116 (11.1%)	119 (11.4%)	111 (10.6%)	147 (14.1%)	617 (11.8%)
1–6 drinks/week	300 (28.8%)	296 (28.4%)	314 (30.1%)	319 (30.6%)	304 (29.1%)	1,533 (29.4%)
1 + drinks/day	125 (12.0%)	142 (13.6%)	132 (12.7%)	125 (12.0%)	113 (10.8%)	637 (12.2%)
Missing	0 (0%)	0 (0%)	2 (0.2%)	2 (0.2%)	0 (0%)	4 (0.1%)
Baseline history of diabetes						
No	1,007 (96.5%)	1,003 (96.2%)	1,012 (97.1%)	1,009 (96.7%)	1,017 (97.5%)	5,048 (96.8%)
Yes	36 (3.5%)	39 (3.7%)	30 (2.9%)	34 (3.3%)	26 (2.5%)	165 (3.2%)
Missing	0 (0%)	1 (0.1%)	0 (0%)	0 (0%)	0 (0%)	1 (0.0%)
Baseline history of hypertension						
No	631 (60.5%)	624 (59.8%)	643 (61.7%)	633 (60.7%)	636 (61.0%)	3,167 (60.7%)
Yes	412 (39.5%)	418 (40.1%)	399 (38.3%)	409 (39.2%)	407 (39.0%)	2,045 (39.2%)
Missing	0 (0%)	1 (0.1%)	0 (0%)	1 (0.1%)	0 (0%)	2 (0.0%)
Baseline treatment of high blood pressure						
No	812 (77.9%)	812 (77.9%)	816 (78.3%)	816 (78.2%)	822 (78.8%)	4,078 (78.2%)
Yes	230 (22.1%)	231 (22.1%)	225 (21.6%)	227 (21.8%)	220 (21.1%)	1,133 (21.7%)
Missing	1 (0.1%)	0 (0%)	1 (0.1%)	0 (0%)	1 (0.1%)	3 (0.1%)
Baseline history of hypercholesterolemia (cholesterol 240+)						
No	578 (55.4%)	589 (56.5%)	596 (57.2%)	585 (56.1%)	600 (57.5%)	2,948 (56.5%)
Yes	464 (44.5%)	453 (43.4%)	446 (42.8%)	457 (43.8%)	443 (42.5%)	2,263 (43.4%)
Missing	1 (0.1%)	1 (0.1%)	0 (0%)	1 (0.1%)	0 (0%)	3 (0.1%)
Baseline treatment of hypercholesterolemia						
No	976 (93.6%)	965 (92.5%)	978 (93.9%)	981 (94.1%)	981 (94.1%)	4,881 (93.6%)
Yes	66 (6.3%)	78 (7.5%)	62 (6.0%)	60 (5.8%)	60 (5.8%)	326 (6.3%)
Missing	1 (0.1%)	0 (0%)	2 (0.2%)	2 (0.2%)	2 (0.2%)	7 (0.1%)

*BMI* Body Mass Index (calculated as weight in kilograms divided by height in meters squared), *LPVN* Licensed Practical Vocational Nurse; global cognitive performance change thresholds: 1st quintile: (-4.682, -0.523), 2nd quintile: (-0.523, -0.126), 3rd quintile: (-0.126, 0.182), 4th quintile: (0.182; 0.562), 5th quintile: (0.562-2.758)

In total, 5,226 of the women participating in the WHS cognitive cohort completed the last cognitive assessment four years after the first assessment. In comparison to women who completed the last assessment, the non-participating women were more likely to be current smokers, to rarely or never engage in strenuous physical activity, and to have a history of diabetes, hypertension, and use of antihypertensive medications, but were less likely to drink alcohol. (see Table A1). On average, women who did not participate in the last cognitive assessment performed slightly worse across all individual test procedures during the first cognitive interview compared with those who completed the last assessment (see Table A2).

We present information on women’s test performance at first and last cognitive interviews for each of the administered test procedures according to cognitive change quintile in Table A3. Women in the 1st and 2nd quintile experienced a decline in cognitive performance over time, whereas those in the 4th and 5st quintile demonstrated improvements. The cut-offs in global cognitive performance change for quintile assignment were the following: 1st quintile: (-4.682, -0.523), 2nd quintile: (-0.523, -0.126), 3rd quintile: (-0.126, 0.182), 4th quintile: (0.182, 0.562), 5th quintile: (0.562, 2.758).

### Primary analysis

The mean follow-up time was 13.4 years (median: 14.4; interquartile range: 10.2–17.8; sum: 70,030.2). Over the period under observation, 3,333 deaths (63.9% of the cohort) were observed.

The assessment of the proportional hazards assumption by means of visually inspecting Kaplan-Meier curves did not result in violations of the former (eFigure 3). A statistically significant shift in all-cause mortality probability between the groups compared was detected by using the log-rank test (*p*-value < 0.001).

**Fig. 1:**
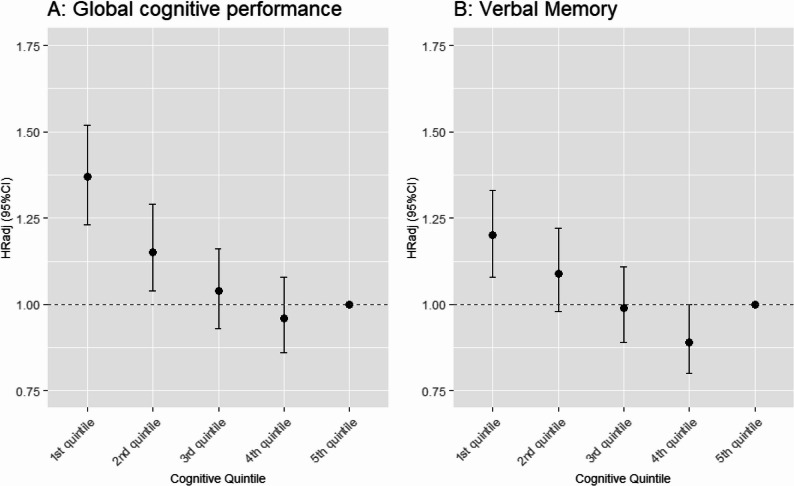
Adjusted HRs with 95% CIs for the risk of all-cause mortality according to cognitive performance change Global cognitive change quintiles (**A**) and verbal memory change quintiles (**B**) and risk for all-cause mortality

Using the 5th quintile with the greatest improvement as the reference, adjusted HR over follow-up time (HR_adj_) for all-cause mortality were 0.96 (95%CI [0.86–1.08]) for the 4th quintile, steadily increasing to 1.04 (95%CI [0.93–1.16]) for the 3rd quintile, 1.15 (95%CI [1.04–1.29]) for the 2nd and 1.37 (95%CI [1.23–1.52]) for the 1st quintile with the greatest decline (Table 2; Fig. 1, Panel A).

**Table 2:** HRs for all-cause mortality according to cognitive performance change quintiles in the WHS cognitive cohort

Global cognitive performance quintiles:	1st quintile (*n* = 1,043) HR (95%CIs)	2nd quintile (*n* = 1,043) HR (95%CIs)	3rd quintile (*n* = 1,042) HR (95%CIs)	4th quintile (*n* = 1,043) HR (95%CIs)	5th quintile (*n* = 1,043) HR [reference]
All-cause mortality, *N*	749	693	647	608	636
Total person-years Rate per 1000 person-years	12,597.5 59.5 deaths/1,000 person-years	13,938.3 49.7 deaths/1,000 person-years	14,181.5 45.6 deaths/1,000 person-years	14,682.5 41.2 deaths/1,000 person-years	14,630.4 43.5 deaths/1,000 person-years
unadjusted	1.52 (1.37–1.69)	1.20 (1.08–1.34)	1.07 (0.96–1.19)	0.95 (0.85–1.06)	1 (ref)
adjusted^1^	1.37 (1.23–1.52)	1.15 (1.04–1.29)	1.04 (0.93–1.16)	0.96 (0.86–1.08)	1 (ref)
Verbal memory performance quintiles:	(*n* = 1,067)	(*n* = 1,025)	(*n* = 1,036)	(*n* = 1,046)	(*n* = 1,040)
All-cause mortality, *N*	738	678	647	605	665
Total person-years Rate per 1000 person-years	13,241.5 55.7 deaths/1,000 person-years	13,640.2 49.7 deaths/1,000 person-years	14,038.7 46.1 deaths/1,000 person-years	14,897.0 40.6 deaths/1,000 person-years	14,213.4 46.8 deaths/1,000 person-years
unadjusted	1.28 (1.16–1.43)	1.09 (0.98–1.21)	1.00 (0.90–1.11)	0.86 (0.77–0.95)	1 (ref)
adjusted^1^	1.20 (1.08–1.33)	1.09 (0.98–1.22)	0.99 (0.89–1.11)	0.89 (0.80-1.00)	1 (ref)

Abbreviations: *CI* Confidence Interval, *HR* Hazard Ratio, *ref* reference group

^1^Adjusted for age, strenuousphysical activity, alcohol use, smoking status, BMI (calculated as weight in kilograms divided by height in meters squared), highest attained educational level, high blood pressure, treatment of high blood pressure, high cholesterol, treatment of high cholesterol, and diabetes at baseline

Analyses additionally adjusting for annual household income yielded similar results to the primary analysis (results not shown).

The effect estimates resulting from the multiple imputation analysis were nearly identical with no meaningful differences in magnitude or statistical significance.

The between-group difference in RMST was  -1.82 ((95%CI -2.21 to -1.43); *p* < 0.001), indicating a reduced average survival time up to 19.9 years among individuals in the lowest 20% of the cognitive decline distribution. The RMST ratio was 0.87 (95%CI [0.85–0.90]).

### Secondary analyses

The estimated average HR_adj_ of all-cause mortality for the 1st quintile when compared to the remaining global cognitive change distribution was 1.32 (95%CI [1.21–1.43]) over the follow-up, further indicating an increased risk of all-cause mortality for women with poorer cognitive performance. Those women in the bottom 10% (*n* = 522; 395 deaths; cut-off = -0.831) of the global cognitive change distribution had a further increased risk for all-cause mortality (HR_adj_ = 1.44 (95%CI [1.29–1.60])) compared to the rest of the cohort.

Similar patterns were observed for verbal memory. Compared to the 5th quintiles, the respective average HR_adj_ were 0.89 (95%CI [0.80–1.00]) for the 4th, 0.99 (95%CI [0.89–1.11]) for the 3rd, 1.09 (95%CI [0.98–1.22]) for the 2nd, and 1.20 (95%CI [1.08–1.33]) for the 1st quintile i.e., the quintile with the greatest decline in verbal memory performance (Fig. 1, Panel B).

## Discussion

In this large prospective cohort of older women in the United States followed for an average of 13 years after cognitive assessment, women with the largest declines in global cognitive function were at increased risk of all-cause mortality when compared to women who cognitively improved over time. This finding was supported when comparing the worst 20% and worst 10% of the cognitive change distribution against the rest of our study sample, as well as for changes in verbal memory.

Impairment or decline in cognitive function has been previously described as being associated with increased risk of all-cause mortality, although the underlying mechanisms remain not fully understood [6–9, 13–15, 33, 34]. Reduced cognitive function may limit the ability to maintain a healthy lifestyle or adhere to medical advice. Cognitive impairment is also a component of broader syndromes such as frailty or dementia, which are themselves linked to higher mortality risk [10–12]. Cognitive decline and mortality may also share underlying pathophysiologic mechanisms. For example, poor vascular health may lead to both cognitive decline (through mechanisms such as microinfarcts, microbleeds, or white matter lesions) and to fatal cardiovascular and cerebrovascular disease events [35–40].

### Comparison with other studies

A meta-analysis examining the sex-specific association between cognitive impairment and all-cause mortality reported that older women with cognitive impairment assessed had a higher mortality risk (RR: 1.48; 95% CI: [1.36–1.61]) compared to men (RR: 1.34; 95% CI: [1.24–1.44]). Most studies included in the meta-analysis assessed cognitive function at a single time point and only used the MMSE, which primarily captures global cognitive performance, to classify participants as cognitively impaired or not [6].

Longitudinal data on cognitive decline remain scarce. One large study from China, including 11,732 older adults, found a monotonic association between MMSE decline and increased 3-year mortality. This association was particularly apparent among those with “*rapid decline*”, defined as those with change greater than the median change among those showing decline (HR: 1.75; 95% CI: [1.57–1.95]) [9]. A few studies have considered alternative tools to assess cognitive performance, addressing not only global cognition, but also specific cognitive domains other or additional instruments to the MMSE. One study using the COGTEL score [41], which includes memory, verbal fluency, and working memory components, found no association between low COGTEL scores and all-cause mortality in women (HR: 1.34 [95%CI (0.77–2.31)]) [13].

In contrast, other studies have observed associations between cognitive impairment and mortality among women. For example, women with cognitive impairment indicated by higher Short Portable Mental Status Questionnaire (SPMSQ) scores, a tool that evaluates orientation, memory, awareness of current events, and basic numerical skills [42], faced a greater risk of all-cause mortality [43]. The Kaunas HAPIEE (Health, Alcohol, and Psychosocial Factors in Eastern Europe) study, examined baseline cognitive performance by means of a cognition battery composed of five tests assessing immediate and delayed verbal memory, semantic verbal fluency, numerical ability, as well as speed and concentration ability and observed associations between lower cognitive performance and increased risk of mortality among women during 10 years of follow-up [8].

In contrast to the aforementioned and most of the other studies in the field, cognitive function in our study was not assessed at a single time point but longitudinally over an approximately four-year follow-up period, thereby capturing change in cognitive performance rather than static measures of cognitive function at a single point in time.

The variations in findings may reflect variations in the cognitive domains captured by the assessment tools used, their sensitivity, or potential ceiling effects. Standardization of cognitive testing or the strategic use of complementary instruments may help improve comparability across studies and increase sensitivity to early decline. Also, different lengths of follow-up or the age at which individuals are followed could complicate direct comparison between studies as mortality risk varies by age group.

Our findings add to existing evidence by showing that even relatively modest declines in cognitive function over time were associated with increased all-cause mortality in women aged 65 years and older. This highlights that mortality risk may increase even before clinical thresholds for impairment are met, suggesting the potential for earlier identification of at-risk individuals. Focusing on decline rather than impairment could consequently help to identify individuals at risk earlier in the disease process.

### Strengths and limitations

Some limitations of our study warrant consideration. Although the overall magnitude of cognitive change observed in this cohort was modest, we observed an increased risk of all-cause mortality among women with declining cognitive performance. Notably, a substantial proportion of participants showed stable or even slightly improved scores over time (4th and 5th quintile). These small improvements may reflect, at least in part, a learning or retest effect rather than true cognitive gains.

As with all observational research, residual and unmeasured confounding cannot be excluded. For example, factors such as depressive symptoms, subclinical cerebrovascular disease, or medication use, which we did not have data on, may have introduced confounding.

In addition, covariate data were collected at baseline and not updated during follow-up so that we were not able to control our analyses for time-varying confounding. Further, cognitive change was assessed between two time points which were about four years apart and women were followed up for the outcome of interest thereafter limiting our ability to account for more complex trajectories of cognitive change the entire follow-up period. Considering the potential influence of more complex trajectories of change warrants further exploration. Also, we were not able to evaluate cognitive change and cause-specific mortality due to changes in the adjudication processes over time.

Due to the requirement that women had to complete both cognitive assessments to be included into our analyses, selection bias cannot be ruled out. However, baseline characteristics and performance in the first cognitive assessment did not differ substantially between women who were later lost to follow-up and those who participated in the last cognitive assessment.

Moreover, our study population was homogeneous as participating women were employed in the health sector and were predominantly white. Thus, the generalizability of our findings to other settings may be limited. However, the homogeneity within our study population may have reduced confounding, specifically regarding access to the healthcare system and its services.

The primary strengths of this study include the standardized assessment of both exposure and outcome, as well as the use of multiple cognitive tests administered at different time points, which enabled the evaluation of changes in test performance over time. Furthermore, the study benefited from a low proportion of missing data for key covariates included in the adjusted models and a comparatively long follow-up period.

In a broader context, our study contributes to the external validation and generalizability of previous findings, as the association between cognitive decline and all-cause mortality has not been well explored specifically within the United States. However, future studies should aim to explicitly account for social and cultural determinants, as they may contribute to the observed relationship.

## Conclusion

In this large prospective cohort of United States-based elderly women, currently or formerly employed in health care professions, cognitive decline was associated with an increase in risk of all-cause mortality. These findings underscore the importance of monitoring cognitive function in the elderly. Further studies are warranted to determine whether modifiable risk factors can be targeted to improve cognitive trajectories and associated health outcomes.

## Supplementary Information

Supplementary Material 1.

## Data Availability

The analyzed data will not be publicly accessible due to protecting the confidentiality and informed consent of the participating women.

## Abbreviations

AD: Alzheimer’s Disease
CI: Confidence Interval
CVD: Cardiovascular Disease
GVIF: Generalized Variance Inflation Factor
HAPIEE: Health, Alcohol, and Psychosocial Factors in Eastern Europe
HR: Hazard Ratio
MMSE: Mini Mental State Examination
MO: Month
OR: Odds Ratio
RMST: Restricted Mean Survival Time
RR: Relative Risk
SPMSQ: Short Portable Mental Status Questionnaire
US: United States
WHS: Women’s Health Study
YR: Year
