# Supplementary material for: Cognitive decline and risk of all-cause mortality in older women: a cohort study
Source: BMC Geriatr. 2026 May 13;26:672. doi: 10.1186/s12877-026-07641-1 (PMC13173952; doi:10.1186/s12877-026-07641-1)
Supplement: Supplementary file 1 — Supplementary Material 1. [file 12877_2026_7641_MOESM1_ESM.docx]

**SUPPLEMENTARY MATERIAL**

**Table A1:** Baseline characteristics of women lost to follow-up and those completing the last cognitive assessment.

|  | **Completed last assessment** (*n* = 5,226) | | **Lost to follow-up** (*n* = 1,151) | | **Overall** (*N* = 6,377) | |  |
| --- | --- | --- | --- | --- | --- | --- | --- |
| **Age** |  | |  | |  | |  |
| Mean (SD) | 66.1 (3.95) | | 66.9 (4.53) | | 66.2 (4.07) | |  |
| Median [Min, Max] | 65.4 [60.4, 87.1] | | 66.0 [60.4, 89.9] | | 65.5 [60.4, 89.9] | |  |
| **BMI** |  | |  | |  | |  |
| Mean (SD) | 25.8 (4.44) | | 25.4 (4.50) | | 25.7 (4.46) | |  |
| Median [Min, Max] | 25.1 [15.7, 51.5] | | 24.6 [14.3, 47.3] | | 25.0 [14.3, 51.5] | |  |
| Missing | 1 (0.0%) | | 4 (0.3%) | | 5 (0.1%) | |  |
| **Highest attained education** |  | |  | |  | |  |
| LPN/LVN, associate’s degree, registered nurse | 3,414 (65.3%) | | 804 (69.9%) | | 4,218 (66.1%) | |  |
| Bachelor’s degree or higher education | 1,728 (33.1%) | | 337 (29.3%) | | 2,065 (32.4%) | |  |
| Missing | 84 (1.6%) | | 10 (0.9%) | | 94 (1.5%) | |  |
| **Smoking status** |  | |  | |  | |  |
| never | 2,765 (52.9%) | | 576 (50.0%) | | 3,341 (52.4%) | |  |
| past | 1,980 (37.9%) | | 415 (36.1%) | | 2,395 (37.6%) | |  |
| current | 475 (9.1%) | | 160 (13.9%) | | 635 (10.0%) | |  |
| Missing | 6 (0.1%) | | 0 (0%) | | 6 (0.1%) | |  |
| **Strenuous physical activity** |  | |  | |  | |  |
| rarely/never | 2,220 (42.5%) | | 531 (46.1%) | | 2,751 (43.1%) | |  |
| <1 time/week | 862 (16.5%) | | 179 (15.6%) | | 1,041 (16.3%) | |  |
| 1 time per week | 432 (8.3%) | | 89 (7.7%) | | 521 (8.2%) | |  |
| 2-3 times/week | 1,093 (20.9%) | | 206 (17.9%) | | 1,299 (20.4%) | |  |
| ≥ 4 times/week | 614 (11.7%) | | 146 (12.7%) | | 760 (11.9%) | |  |
| Missing | 5 (0.1%) | | 0 (0%) | | 5 (0.1%) | |  |
| **Alcohol use** |  | |  | |  | |  |
| Rarely/never | 2,431 (46.5%) | | 608 (52.8%) | | 3,039 (47.7%) | |  |
| 1-3 drinks/month | 618 (11.8%) | | 126 (10.9%) | | 744 (11.7%) | |  |
| 1-6 drinks/week | 1,535 (29.4%) | | 277 (24.1%) | | 1,812 (28.4%) | |  |
| 1+ drinks/day | | 638 (12.2%) | | 140 (12.2%) | | 778 (12.2%) | |
| Missing | | 4 (0.1%) | | 0 (0%) | | 4 (0.1%) | |
| **Baseline history of diabetes** | |  | |  | |  | |
| No | | 5,060 (96.8%) | | 1,093 (95.0%) | | 6,153 (96.5%) | |
| Yes | | 165 (3.2%) | | 58 (5.0%) | | 223 (3.5%) | |
| Missing | | 1 (0.0%) | | 0 (0%) | | 1 (0.0%) | |
| **Baseline history of hypertension** | |  | |  | |  | |
| No | | 3,171 (60.7%) | | 649 (56.4%) | | 3,820 (59.9%) | |
| Yes | | 2,053 (39.3%) | | 502 (43.6%) | | 2,555 (40.1%) | |
| Missing | | 2 (0.0%) | | 0 (0%) | | 2 (0.0%) | |
| **Baseline treatment of high blood pressure** | |  | |  | |  | |
| No | | 4,087 (78.2%) | | 855 (74.3%) | | 4,942 (77.5%) | |
| Yes | | 1,136 (21.7%) | | 291 (25.3%) | | 1,427 (22.4%) | |
| Missing | | 3 (0.1%) | | 5 (0.4%) | | 8 (0.1%) | |
| **Baseline history of hypercholesterolemia (cholesterol 240+)** | |  | |  | |  | |
| No | | 2,955 (56.5%) | | 680 (59.1%) | | 3,635 (57.0%) | |
| Yes | | 2,268 (43.4%) | | 471 (40.9%) | | 2,739 (43.0%) | |
| Missing | | 3 (0.1%) | | 0 (0%) | | 3 (0.0%) | |
| **Baseline treatment of hypercholesterolemia** | |  | |  | |  | |
| No | | 4,891 (93.6%) | | 1,082 (94.0%) | | 5,973 (93.7%) | |
| Yes | | 328 (6.3%) | | 69 (6.0%) | | 397 (6.2%) | |
| Missing  **BMI**: Body Mass Index (calculated as weight in kilograms divided by height in meters squared); **LPVN**: Licensed Practical Vocational Nurse | | 7 (0.1%) | | 0 (0%) | | 7 (0.1%) | |

**Table A2:** Performance of women in the WHS cognitive cohort at first cognitive assessment by follow-up status.

| **First assessment** | **Completed last assessment** (*N* = 5,226) | **Lost to follow-up** (*N* = 1,151) | **Overall** (*N* = 6,377) |
| --- | --- | --- | --- |
| **TICS**^1^ |  |  |  |
| Mean (SD) | 34.4 (2.54) | 33.5 (3.39) | 34.2 (2.73) |
| Median [Min, Max] | 35.0 [21.0, 41.0] | 34.0 [4.00, 41.0] | 34.0 [4.00, 41.0] |
| Missing | 0 (0%) | 15 (1.3%) | 15 (0.2%) |
| **Immediate East Boston Memory Test**^2^ |  |  |  |
| Mean (SD) | 9.65 (1.56) | 9.25 (1.68) | 9.58 (1.59) |
| Median [Min, Max] | 10.0 [0, 12.0] | 10.0 [0, 12.0] | 10.0 [0, 12.0] |
| Missing | 1 (0.0%) | 4 (0.3%) | 5 (0.1%) |
| **Delayed East Boston Memory Test^2^** |  |  |  |
| Mean (SD) | 9.36 (1.67) | 8.90 (2.20) | 9.28 (1.79) |
| Median [Min, Max] | 10.0 [0, 12.0] | 9.00 [0, 12.0] | 10.0 [0, 12.0] |
| Missing | 0 (0%) | 16 (1.4%) | 16 (0.3%) |
| **Immediate 10 word list**^3^ |  |  |  |
| Mean (SD) | 4.87 (1.73) | 4.63 (1.92) | 4.83 (1.77) |
| Median [Min, Max] | 5.00 [0, 10.0] | 4.00 [0, 10.0] | 5.00 [0, 10.0] |
| Missing | 1 (0.0%) | 12 (1.0%) | 13 (0.2%) |
| **Delayed 10 word list**^3^ |  |  |  |
| Mean (SD) | 3.04 (2.04) | 2.71 (2.33) | 2.98 (2.10) |
| Median [Min, Max] | 3.00 [0, 10.0] | 2.00 [0, 10.0] | 3.00 [0, 10.0] |
| Missing | 0 (0%) | 16 (1.4%) | 16 (0.3%) |
| **Category fluency test^4^** |  |  |  |
| Mean (SD) | 17.8 (4.87) | 16.1 (5.07) | 17.5 (4.95) |
| Median [Min, Max] | 18.0 [0, 43.0] | 16.0 [0, 34.0] | 17.0 [0, 43.0] |
| Missing | 1 (0.0%) | 23 (2.0%) | 24 (0.4%) |

^1^ Telephone Interview of Cognitive Status (TICS) (max. 41 points)

^2^ Max. 12 points

^3^ Max. 10 points

^4^ Women were asked to name as many animals as possible in one minute

**Table A3:** Performance at first and last assessment by global cognitive quintiles.

|  | 1^st^ quintile (*n* = 1,043) | 2^nd^ quintile (*n* = 1,043) | 3^rd^ quintile (*n* = 1,042) | 4^th^ quintile (*n* = 1,043) | 5^th^ quintile (*n* = 1,043) | Overall (*N* = 5,214) |
| --- | --- | --- | --- | --- | --- | --- |
| **TICS**^1^ |  |  |  |  |  |  |
| **First assessment** |  |  |  |  |  |  |
| Mean (SD) | 34.8 (2.51) | 34.7 (2.49) | 34.4 (2.42) | 34.3 (2.41) | 33.6 (2.59) | 34.4 (2.52) |
| Median [Min, Max] | 35.0 [23.0, 41.0] | 35.0 [25.0, 41.0] | 35.0 [24.0, 41.0] | 34.0 [23.0, 41.0] | 34.0 [21.0, 41.0] | 35.0 [21.0, 41.0] |
| **Last assessment** |  |  |  |  |  |  |
| Mean (SD) | 32.3 (3.40) | 33.7 (2.68) | 34.3 (2.57) | 34.9 (2.48) | 35.9 (2.61) | 34.2 (3.02) |
| Median [Min, Max] | 33.0 [12.0, 40.0] | 34.0 [19.0, 41.0] | 34.0 [24.0, 41.0] | 35.0 [23.0, 41.0] | 36.0 [24.0, 41.0] | 34.0 [12.0, 41.0] |
| **Immediate East Boston Memory Test**^2^ |  |  |  |  |  |  |
| **First assessment** |  |  |  |  |  |  |
| Mean (SD) | 10.1 (1.48) | 9.99 (1.42) | 9.77 (1.52) | 9.56 (1.47) | 8.80 (1.55) | 9.65 (1.56) |
| Median [Min, Max] | 10.0 [5.00, 12.0] | 10.0 [4.00, 12.0] | 10.0 [3.00, 12.0] | 10.0 [3.00, 12.0] | 9.00 [0, 12.0] | 10.0 [0, 12.0] |
| **Last assessment** |  |  |  |  |  |  |
| Mean (SD) | 8.17 (1.68) | 9.34 (1.59) | 9.84 (1.55) | 10.3 (1.53) | 10.6 (1.42) | 9.65 (1.77) |
| Median [Min, Max] | 8.00 [0, 12.0] | 10.0 [2.00, 12.0] | 10.0 [4.00, 12.0] | 10.0 [3.00, 12.0] | 11.0 [6.00, 12.0] | 10.0 [0, 12.0] |
| **Delayed East Boston Memory Test**^2^ |  |  |  |  |  |  |
| **First assessment** |  |  |  |  |  |  |
| Mean (SD) | 9.71 (1.75) | 9.65 (1.55) | 9.57 (1.54) | 9.31 (1.58) | 8.56 (1.65) | 9.36 (1.67) |
| Median [Min, Max] | 10.0 [0, 12.0] | 10.0 [0, 12.0] | 10.0 [0, 12.0] | 10.0 [0, 12.0] | 8.00 [0, 12.0] | 10.0 [0, 12.0] |
| **Last assessment** |  |  |  |  |  |  |
| Mean (SD) | 7.53 (2.48) | 9.11 (1.67) | 9.53 (1.59) | 10.0 (1.62) | 10.3 (1.47) | 9.30 (2.05) |
| Median [Min, Max] | 8.00 [0, 12.0] | 9.00 [0, 12.0] | 10.0 [0, 12.0] | 10.0 [0, 12.0] | 10.0 [0, 12.0] | 10.0 [0, 12.0] |
| **Delayed 10 word list**^3^ |  |  |  |  |  |  |
| **First assessment** |  |  |  |  |  |  |
| Mean (SD) | 3.39 (2.32) | 3.33 (2.11) | 3.00 (1.98) | 2.94 (1.84) | 2.57 (1.80) | 3.05 (2.04) |
| Median [Min, Max] | 3.00 [0, 10.0] | 3.00 [0, 10.0] | 3.00 [0, 10.0] | 3.00 [0, 10.0] | 2.00 [0, 10.0] | 3.00 [0, 10.0] |
| **Last assessment** |  |  |  |  |  |  |
| Mean (SD) | 2.20 (1.92) | 2.99 (2.06) | 3.36 (2.03) | 3.80 (2.08) | 4.76 (2.40) | 3.42 (2.27) |
| Median [Min, Max] | 2.00 [0, 10.0] | 3.00 [0, 10.0] | 3.00 [0, 10.0] | 4.00 [0, 10.0] | 4.00 [0, 10.0] | 3.00 [0, 10.0] |
| **Category fluency test**^4^ |  |  |  |  |  |  |
| **First assessment** |  |  |  |  |  |  |
| Mean (SD) | 18.3 (5.09) | 18.6 (4.97) | 17.9 (4.54) | 17.8 (4.83) | 16.6 (4.67) | 17.8 (4.87) |
| Median [Min, Max] | 18.0 [5.00, 43.0] | 18.0 [4.00, 37.0] | 18.0 [5.00, 35.0] | 18.0 [6.00, 36.0] | 16.0 [0, 33.0] | 18.0 [0, 43.0] |
| **Last assessment** |  |  |  |  |  |  |
| Mean (SD) | 15.5 (4.69) | 17.2 (4.95) | 18.0 (4.76) | 18.9 (5.08) | 19.2 (5.48) | 17.8 (5.17) |
| Median [Min, Max] | 15.0 [4.00, 32.0] | 17.0 [5.00, 38.0] | 18.0 [6.00, 44.0] | 18.0 [5.00, 37.0] | 19.0 [6.00, 49.0] | 17.0 [4.00, 49.0] |
| ***Z*-score difference [global change score]**^5^ |  |  |  |  |  |  |
| Mean (SD) | -0.948 (0.428) | -0.313 (0.113) | 0.0298 (0.0900) | 0.359 (0.108) | 0.938 (0.340) | 0.0130 (0.684) |
| Median [Min, Max] | -0.831 [-4.68, -0.523] | -0.308 [-0.523, -0.126] | 0.0287 [-0.126, 0.182] | 0.351 [0.182, 0.562] | 0.836 [0.562, 2.76] | 0.0287 [-4.68, 2.76] |

^1^ Telephone Interview of Cognitive Status (TICS) (max. 41 points)

^2^ Max. 12 points

^3^ Max. 10 points

^4^ Women were asked to name as many animals as possible in one minute

^5^ Global test scores were generated by averaging Z-scores of the performance in each of the five tests

**
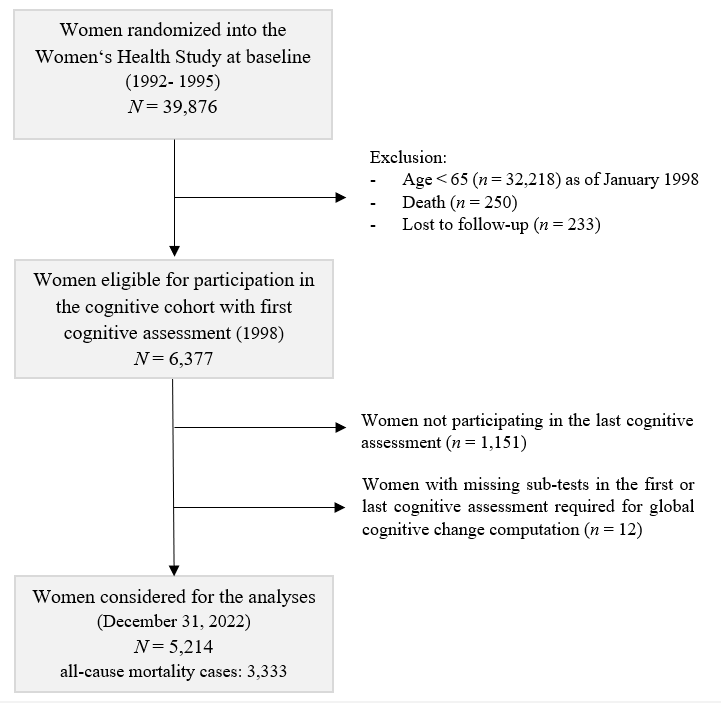
**

**eFigure 1:** Flow chart for inclusion among women participating in the WHS cognitive cohort.


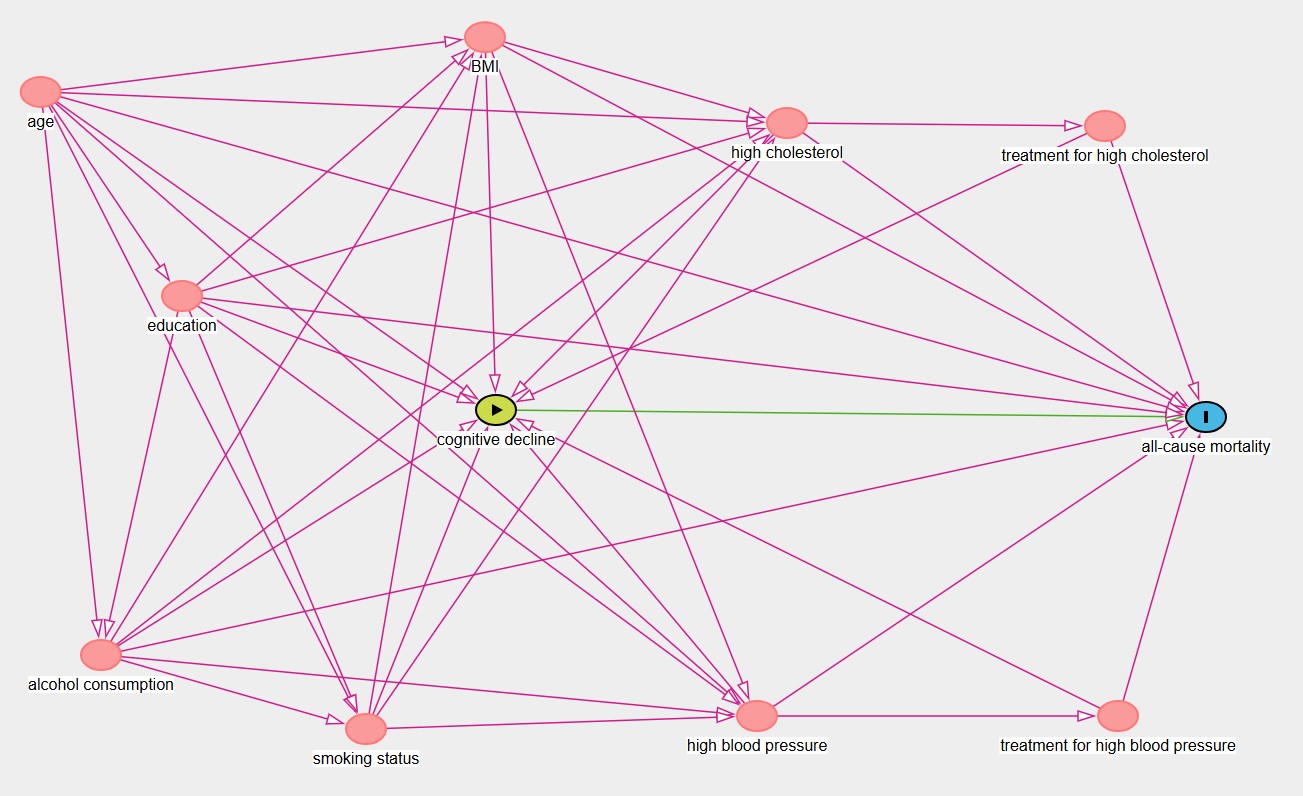


BMI: Calculated as weight in kilograms divided by height in meters squared

**eFigure 2:** Directed acyclic graph for the effect of *“cognitive decline“* on the risk *“all-cause mortality”.*


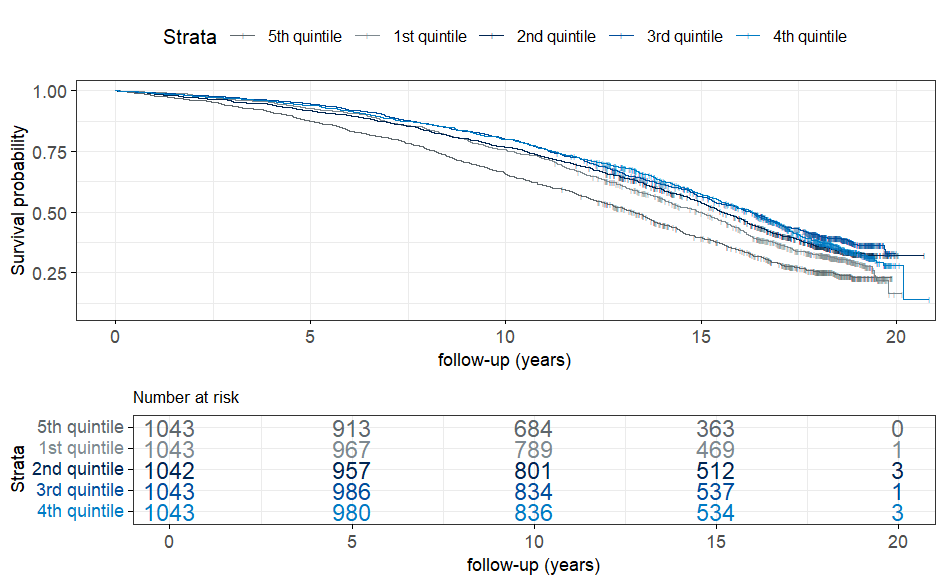


Person-time was calculated from the third cognitive assessment until the date of all-cause death, loss to follow-up, or the end of the study (December 31, 2022), whichever occurred first.

**eFigure 3:** Kaplan Meier curves for the survival probability among women with complete global cognitive performance information (*N* = 5,214).
